# Dietary Antioxidant Vitamins and Minerals and Breast Cancer Risk: Prospective Results from the SUN Cohort

**DOI:** 10.3390/antiox10030340

**Published:** 2021-02-24

**Authors:** Cesar I. Fernandez-Lazaro, Miguel Ángel Martínez-González, Inmaculada Aguilera-Buenosvinos, Alfredo Gea, Miguel Ruiz-Canela, Andrea Romanos-Nanclares, Estefanía Toledo

**Affiliations:** 1 Department of Preventive Medicine and Public Health, University of Navarra, 31008 Pamplona, Spain; cflazaro@unav.es (C.I.F.-L.); mamartinez@unav.es (M.Á.M.-G.); iaguilera@alumni.unav.es (I.A.-B.); ageas@unav.es (A.G.); mcanela@unav.es (M.R.-C.); aromanos@alumni.unav.es (A.R.-N.); 2 IdisNA, Navarra Institute for Health Research, 31008 Pamplona, Spain; 3 CIBERobn Physiopathology of Obesity and Nutrition, Institute of Health Carlos III (ISCIII), 28029 Madrid, Spain; 4 Department of Nutrition, Harvard T.H. Chan School of Public Health, Harvard University, Boston, MA 02115, USA

**Keywords:** breast cancer, antioxidants, vitamin A, vitamin C, vitamin E, selenium, zinc, SUN cohort, Mediterranean population

## Abstract

There is growing interest in natural antioxidants and their potential effects on breast cancer (BC). Epidemiological evidence, however, is inconsistent. We prospectively evaluated the association between dietary intake of vitamins A, C, and E, selenium, and zinc and BC among 9983 female participants from the SUN Project, a Mediterranean cohort of university graduates. Participants completed a food frequency questionnaire at baseline, and biennial follow-up information about incident BC diagnosis was collected. Cases were ascertained through revision of medical charts and consultation of the National Death Index. Cox proportional hazards models were used to estimate multivariable-adjusted hazard ratios (HRs) and 95% confidence intervals (CI). During an average follow-up of 11.3 years, 107 incident BC cases were confirmed. The multivariable HRs (95% CI) for BC comparing extreme tertiles of energy-adjusted dietary intakes were 1.07 (0.64–1.77; *P*_trend_ = 0.673) for vitamin A, 1.00 (0.58–1.71; *P*_trend_ = 0.846) for vitamin C, 0.92 (0.55–1.54; *P*_trend_ = 0.728) for vitamin E, 1.37 (0.85–2.20; *P*_trend_ = 0.135) for selenium, and 1.01 (0.61–1.69; *P*_trend_ = 0.939) for zinc. Stratified analyses showed an inverse association between vitamin E intake and postmenopausal BC (HR_T3 vs. T1_ = 0.35; 95% CI, 0.14–0.86; *P*_trend_ = 0.027). Our results did not suggest significant protective associations between dietary vitamins A, C, and E, selenium, or zinc and BC risk.

## 1. Introduction

Cancer is currently the most common cause of premature mortality in most developed countries and ranks second in terms of global mortality, accounting for 9.6 million deaths in 2018 [1,2]. Moreover, the incidence of cancer is expected to increase as the population ages [3]. In 2018, cancer was the leading cause of death in Spain for both men and women, with 297.8 and 186.7 deaths per 100,000 inhabitants, respectively [4]. Genetic predisposition is a well-known risk factor but it is estimated that the contribution of genetic factors on cancer risk is approximately between 5% and 10%, whereas environmental and lifestyle factors may account for the remaining 90–95% of cases [5]. Among environmental factors, cumulative lifetime exposure to oxidative damage has been suggested to be involved in both cancer initiation and progression [6]. Diet, a potentially modifiable lifestyle risk factor, may contribute up to 35% of cancer cases, which highlights opportunities for cancer incidence prevention [5]. 

Extensive and robust evidence has demonstrated in the last few decades that diet plays a direct role in the development of certain types of cancer, such as breast cancer (BC) [7,8,9]. In this context, natural compounds in the diet, including vitamins and minerals, have been postulated as anticarcinogenic agents due to their antioxidant properties [10,11]. These micronutrients prevent an excess of reactive oxygen species (ROS) and maintain an adequate reduction–oxidation balance. ROS production promotes DNA damage in cancer and genetic instability. Antioxidants can scavenge free radicals and quench the process of lipid peroxidation, which may reduce the oxidative DNA damage caused by free radicals, and ultimately protect against BC [12,13]. Antioxidant vitamins and minerals interrupt free radical chain reactions and operate in the early and late stages of carcinogenesis. Furthermore, antioxidants promote cancer cell death by producing modifications in cell signaling, changes in the cell cycle progression, and modulation of enzymatic activities [14].

Despite the existing evidence on the anticarcinogenic activity of antioxidants in vitro and animal studies, results from epidemiological studies have engaged extensive controversy owing to inconsistent findings [15,16,17,18]. Some studies have reported no clear evidence for the association between dietary antioxidants and BC [15,16,18], whereas few others have suggested possible inverse associations [17,19]. Hence, we aimed to prospectively investigate whether dietary intake of vitamins A, C, and E, selenium, and zinc was associated with risk of overall, pre-, and postmenopausal BC in the SUN (Seguimiento Universidad de Navarra) Study, a Mediterranean cohort of graduates.

## 2. Materials and Methods

### 2.1. Study Population

The SUN Project [20] is a large, prospective, ongoing, and permanently open cohort of Spanish university graduates. The design, objectives, and methods of the SUN cohort have been described elsewhere [21]. Briefly, the cohort aims to assess the role of diet and lifestyle on chronic disease prevention and mortality. Participant recruitment started in December 1999, and follow-up information has been biennially assembled using mailed or online questionnaires. 

**Figure 1:**
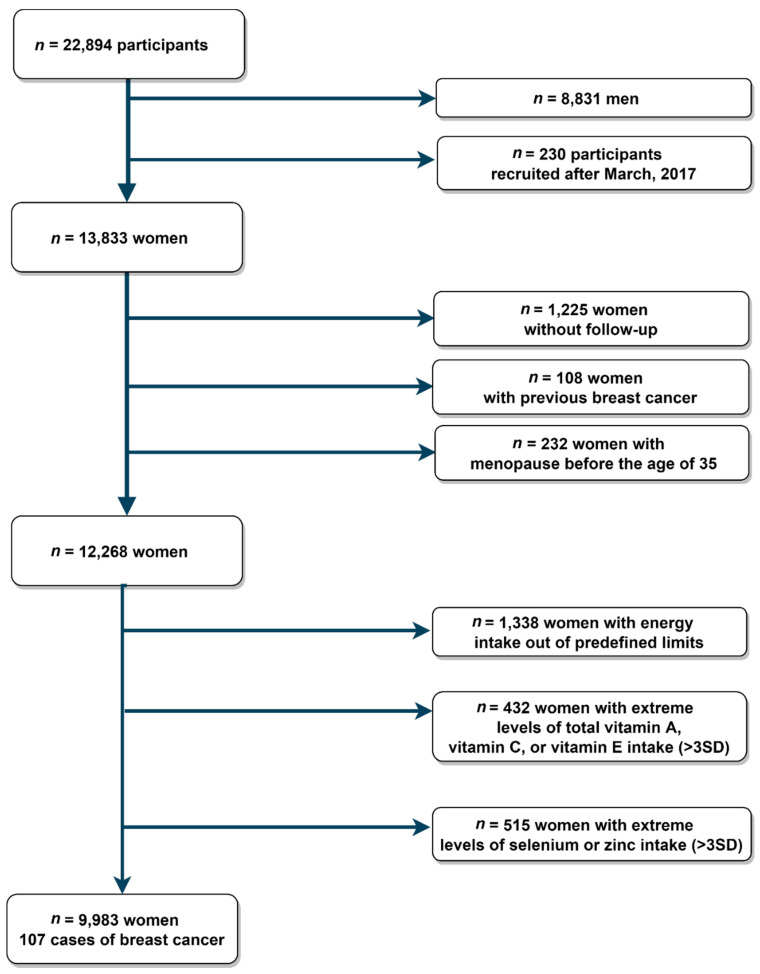
Flowchart of study participants for the assessment of the association of vitamin A, vitamin C, vitamin E, selenium, and zinc and incident intake and breast cancer in the SUN (University of Navarra Follow-up) Project (1999–2020).

By December 2019, a total of 22,894 participants had completed the baseline questionnaire. For the analysis of the present study, we excluded men (*n* = 8831) and participants recruited after March 2017 (*n* = 230). This exclusion was performed to guarantee a follow-up time of at least two years. Among the remaining female participants, we excluded 1225 participants without follow-up (overall retention 91%), 108 women with previous BC history, and 232 participants who reported menopause before the age of 35 years. We additionally excluded 1338 women with daily energy intake out of predefined limits (<500 or >3500 kcal/d) [22] and participants who reported extreme levels (> 3 standard deviations [SD]) of vitamins A, C, and E, selenium, or zinc intake (*n* = 947). The final sample for the present study analyses consisted of 9983 female participants (Figure 1).

### 2.2. Exposure Assessment

Dietary intake information was assessed at baseline with a 136-item semi-quantitative food frequency questionnaire (FFQ) previously validated in Spain [23,24,25]. Validation of the FFQ with repeated 3-day dietary records showed an intra-class correlation coefficient (ICC) = 0.84 for vitamin A, ICC = 0.87 for vitamin C, and ICC = 0.75 for vitamin E [25]. Participants reported their average frequency of food consumption (nine categories ranging from “never” to “more than six times per day”) in common serving size. Food composition tables were used to calculate energy and nutrient intakes for each participant [26,27]. The nutrient contribution of each food item was calculated by multiplying the frequency of food consumption by the nutrient composition of the specified portion size. Nutrients were adjusted for energy intake with the residuals method [22] and categorized into tertiles. 

### 2.3. Incident Breast Cancer Ascertainment 

The primary endpoint of the current study was incident BC. In the biennial follow-up questionnaires, women were asked to self-report any new diagnosis of BC, and medical records were requested in order to confirm the diagnosis. A trained oncologist, blinded to the different exposures, reviewed participants’ medical records, and confirmed the diagnosis. Information on fatal causes was reported to our research team by the subjects’ next of kin, postal authorities, or work associates. Furthermore, we consulted the National Death Index at least once a year to identify deceased cohort members when participants were lost to follow-up, or when we had no information on the death causes. Women for whom the reported cause of death in the National Death Index was BC were treated as confirmed incident cases.

### 2.4. Covariates

At baseline, participants provided information regarding socio-demographic characteristics, anthropometric measures, lifestyle behaviors (i.e., smoking, alcohol intake, physical activity, among others), medical history, and obstetric information (i.e., family history of BC, number of and age at pregnancies, months of breastfeeding, age of menarche, menopausal status, and age at menopause if applicable). Accuracy of self-reported weight and height to calculate body mass index (BMI) was previously validated in a subsample of this cohort [28]. Physical activity information was gathered with a previously validated questionnaire [29]. We estimated metabolic equivalents (METs) for each participant to obtain METs-h/week dedicated to all physical activities performed during leisure time. Adherence to the Mediterranean Diet (MedDiet) was assessed with the score proposed by Trichopoulou et al. [30], excluding alcohol intake. Hence, the MedDiet score ranged from 0 to 8, with higher scores meaning greater adherence. 

### 2.5. Statistical Analysis

Descriptive statistics were used to summarize the characteristics of the study population at baseline. The contribution of each food included in the FFQ to the between-subject variation of dietary exposures intake was estimated by nested regression analyses after a stepwise regression and expressed as cumulative R^2^ change. We additionally calculated the percentage (%) of the contribution of each FFQ item and food groups to the dietary exposure intakes. 

Cox proportional hazard models were performed to examine the association of energy-adjusted tertiles of vitamins A, C, and E, selenium, and zinc intake with BC risk. Results were expressed as hazard ratios (HRs) with corresponding 95% confidence intervals (95% CIs), considering the lowest tertile as the reference category. All models included age as an underlying time variable and were additionally stratified by year of recruitment (four categories) and age at recruitment (decades). Time at entry was the date of completion of the baseline questionnaire; exit time was the age when participants were diagnosed with BC, died, or responded the latest follow-up questionnaire, if they were alive and free of BC at the end of follow-up. After crude analyses, different models were fitted to include potential confounders that may influence the effects of the study exposures on BC risk; model 1 was adjusted for age at menarche (four categories), age at menopause (three categories), alcohol intake (g/d, continuous), breastfeeding (months, continuous), BMI (kg/m^2^, continuous), height (cm, continuous), hormone replacement therapy (dichotomous) and its duration (years, continuous), obstetric history (five categories), physical activity (METs-h/week, continuous), relatives with history of BC (three categories), smoking habit (package/year, continuous), smoking status (three categories), and years at university (continuous); model 2 was additionally adjusted for calcium intake (mg/d, continuous), coffee consumption (two categories), fat intake (E%, continuous), Mediterranean diet adherence (points, continuous), sugar-sweetened beverage consumption (three categories), total energy intake (Kcal/d, continuous), TV-watching (hours/d, continuous), and use of supplements (dichotomous). The confounders of the study were selected considering previously published literature and preceding results of the SUN cohort on BC [31,32,33,34]. We performed tests of linear trend across tertiles of vitamins A, C, and E, selenium, and zinc intake by assigning the median value to each tertile and treating the resulting variables as continuous.

We stratified our analyses by menopausal BC. Information regarding menopause was collected at baseline and after 16 years of follow-up. When this information was not available, age at menopause was considered to be 52 years (percentile 75 of those women who reported age at menopause) [35]. For premenopausal BC as outcome, only women without menopause at baseline were considered, and follow-up time was censored at the age when participants self-reported menopause or at the age of 52 years, whichever happened first. For postmenopausal BC as outcome, we examined women with menopause at baseline or premenopausal women only after their self-reported menopause during the follow-up or after they had turned 52 years during the follow-up, whichever happened last. For premenopausal analyses, models were not adjusted for age at menopause, use of hormone replacement therapy and its duration. For postmenopausal analyses, models were additionally adjusted for time since recruitment until the beginning of the time at risk (years, continuous).

We additionally repeated our analyses considering only luminal BC. As information related to cancer subtype was not available for all the BC incident cases and the number of some BC subtypes was relatively low, we limited our analyses to confirmed luminal BC. 

To assess the robustness of our findings, we performed sensitivity by re-running our models after excluding participants with a follow-up of <2 years, truncating the participants’ follow-up at 10 years, and using an alternative definition of exposure. The exposure was defined as total intake of sources from both diet and supplements, except for selenium supplemental intake which information was not available. For regular multivitamin and supplement users, information regarding dosage, brand, and frequency was collected.

All analyses were performed using Stata software, version 16.0 (Stata Corporation LP, College Station, TX, USA), and a 2-sided *p*-value < 0.05 was deemed as statistically significant. Missing covariate data were imputed with regression equations to predict missing values. Imputations did not imply any missing outcome and represented < 5% of missing covariates.

## 3. Results

During a mean average of 11.3 years of follow-up, 107 incident BC cases were diagnosed in 9983 women. The crude BC incidence was 94.7 per 100,000 person-year. Among the 72 cases with known subtype, 57 cases were luminal (79.2%), 8 cases were HER2 (11.1%), and 7 cases were triple negative (9.7%) tumors. The distribution of the study subjects by baseline characteristics is summarized in Table 1. The mean age of participants was 35.1 years (SD = 10.5 years), most of them were non-smokers, non-users of multivitamin or mineral supplements, had a low consumption of sugar-sweetened beverages, and they were largely nulliparous and mostly premenopausal.

Pearson correlations between dietary intake of antioxidants (non-energy-adjusted) and energy intake were moderate for selenium (*r* = 0.58), vitamin E (*r* = 0.57), and weaker for zinc (*r* = 0.36), vitamin C (*r* = 0.36), and vitamin A (*r* = 0.29). The individual and cumulative contributions of different foods to the variability in antioxidant vitamins and minerals as well as the between-person variability are shown in Supplemental Table S1. In brief, the primary food group contributors of dietary daily intake were vegetables and fruits for vitamins A and C, fats and oils for vitamin E, fish and seafood for selenium, and dairy products for zinc. Regarding sources of variability, fruits and vegetables explained more than 98% of the between-person variability for vitamin A and C intake, while fats and oils explained 43% for vitamin E intake, cereals and legumes 40% of selenium intake, and dairy products represented 82% of the between-person variability for zinc intake.

Hazard ratios and 95% CI for the risk of BC according to dietary intake of vitamin A, vitamin C, and vitamin E among overall, premenopausal, and postmenopausal women are shown in Table 2, Table 3 and Table 4, respectively. Dietary vitamin A, vitamin C, and vitamin E intakes were not significantly associated with overall BC risk (highest vs. lowest tertile): HR, 1.07; 95%CI (0.64–1.77; *P*_trend_ = 0.673) for vitamin A; 1.00 (0.58–1.71; *P*_trend_ = 0.846) for vitamin C; and 0.92 (0.55–1.54; *P*_trend_ = 0.728) for vitamin E. When we stratified by menopausal status, no significant association was observed between tertiles of these antioxidant dietary intakes and BC incidence, except for vitamin E intake. Among postmenopausal women, we found an inverse association between vitamin E intake and BC risk for tertile 3 vs. tertile 1: HR, 0.35; 95% CI (0.14–0.86; *P*_trend_ = 0.027).

Table 5 and Table 6 show the results for multivariable-adjusted models assessing dietary intakes of selenium and zinc with overall BC and among pre- and postmenopausal women. No significant association was observed between antioxidant minerals intake and overall BC, either for premenopausal or postmenopausal BC, across different levels of dietary antioxidant intake. 

When we considered luminal BC as outcome, we did not find associations between levels of antioxidant vitamin or mineral intake and BC risk (Supplemental Table S2).

After re-running the models under different assumptions, no evidence of a clear association was found between antioxidants and BC risk. (Supplemental Tables S3–S5).

## 4. Discussion

In this prospective cohort study of Spanish university graduates, we aimed to prospectively investigate the relationship between the intake of vitamins A, C, and E, selenium, and zinc and BC risk. After multiple adjustments for traditional risk factors, we did not find any evidence of the association between antioxidant vitamins or minerals with overall BC risk. When we stratified our analyses by menopausal status, an inverse association with BC was observed only for vitamin E intake among postmenopausal women. 

A large number of epidemiologic studies have examined for a long period the association of natural antioxidants with BC risk. Findings, however, have been predominantly discordant [15]. Discrepancies in results seem to differ by study design. On the one hand several case-control studies have suggested an inverse relationship between antioxidant vitamins and BC risk. On the other hand, most cohort studies published in the last decade have found no consistent evidence of such association. For example, a recent meta-analysis of observational studies which included the most up-to-date studies on vitamin C intake and BC risk [19] concluded that higher dietary vitamin C intake was significantly associated with a lower BC risk in pooled analyses and case-control studies; nevertheless, no significant observation was observed in subgroup analyses of cohort studies. Similar findings were observed in a previous meta-analysis in which dietary intake of retinol, vitamins A, C, and E became non-significant when data from cohort studies were pooled [16]. Recall and selection bias in case-control studies might explain such inconsistencies between study designs, although no clear explanations exist [36]. Case-control studies are prone to selection bias, particularly diet and cancer studies, due to the difficulty of selecting the appropriate control group [37]. Moreover, recall bias may often occur because cases may associate unhealthy foods and habits with their BC malignancy [15]. 

The present study provides no evidence of an association between antioxidant vitamins and BC, except for vitamin E and postmenopausal women, but the association was restricted to dietary intake. Evidence of supplementation of antioxidant vitamins has not been compelling for BC [38,39], consistent with prior findings from large cohorts. In the European Prospective Investigation into Cancer and Nutrition (EPIC) study, neither dietary vitamin C nor vitamin E was associated with overall BC, and neither were they in analyses stratified by menopausal status [40]. Similarly, a pooled analysis from five established cohorts in the UK Dietary Cohort Consortium study found no evidence of an association between dietary vitamin C intake and BC risk [17]; these findings are consistent with the Swedish Mammography Screening Cohort in which no overall association between intake of vitamin C, beta-carotene, retinol, or vitamin E and BC incidence was found [41]. Only a few observational studies have assessed the relationship between BC risk and dietary selenium and zinc intake, finding no evidence of such association [42,43]. Interestingly, the conclusions from the systematic review and meta-analysis of prospective studies conducted by Kuria et al. [18] suggested a protective effect of a recommended daily allowance of selenium intake for overall cancer; nevertheless, the potential protective effect was non-significant for BC. 

Regarding dietary vitamins and minerals intake, the national recommended dietary allowances intakes for adult women are 600 mcg/d for vitamin A, 60 mg/d for vitamin C, 12 mg/d for vitamin E, 55 mcg/d for selenium, and 7 mg/d for zinc [44]. In our study, median intake in all energy-adjusted antioxidant tertiles was higher than the national recommended dietary allowances, except for vitamin E, for which not even the median intake in the highest tertile met such allowances. The dietary intake levels of vitamin and antioxidants of the present study are higher than the levels reported by the Anibes study, the latest national published research [45]. 

Animal studies have demonstrated the important role of ROS and breast malignancy [46]. Non-enzymatic antioxidants include natural compounds such as vitamins A, C, and E, or minerals such as selenium and zinc, supplied through foods and supplements that help endogenous compounds reduce a variety of ROS. A potential mechanism for inducing tumor reduction is based on the antioxidants’ capacity to control the redox balance in malignant cells [46,47]. Reactive species may cause severe oxidative stress and may lead to DNA damage, suppress tumor genes, and alter cellular homeostasis leading to carcinogenesis [12,13]. Specifically, vitamin A, selenium, and zinc have been hypothesized to diminish the risk of BC by inhibition of cell proliferation, induction of differentiation, and apoptosis [48,49,50]. Vitamin C plays a key role as a prooxidant breakage of cellular DNA [51], and vitamin E additionally suppresses lipid peroxidation and induces apoptosis of tumor cells [52,53]. A major interest has been drawn among postmenopausal women, of whom the consumption of antioxidants has been theorized to have greater benefits than among premenopausal women [54]. While premenopausal women maintain an oxidative balance between ROS and the body’s antioxidant mechanisms, produced mostly by the inhibition of the 8-hydroxylation of guanine DNA bases [55], the levels of estrogens generally decline as menopause nears, leading to increased levels of oxidative stress [56]. Lower levels of estrogens have pro-oxidant effects that may trigger the oxidation of bases, DNA adducts, and genetic material damage [55].

In our study, there was no clear evidence of an association between antioxidants and BC risk. A potential explanation for these results may rely on the bioavailability of antioxidant compounds. Some authors have suggested that not only intake levels but also their bioavailability may influence their health benefits [57]. Furthermore, compounds’ antioxidant activity largely depends on the cooking method, as foods may suffer losses of antioxidant properties [58]. Environmental and cultivation conditions may also play a significant role on the antioxidant activity of certain foods. Different agroclimatic locations as well as factors such as temperature, moisture, and harvesting period may determine the antioxidant content [59]. Assessment of the overall dietary antioxidant capacity rather than individual antioxidant intake has emerged as an alternative approach in the last few years. Similar to the food synergy concept [60], the food antioxidant matrix may be greater than the corresponding action of the individual antioxidants. In other words, the cumulative effects of individual antioxidants may be too small to see a clear effect, and correlations and interactions between the endogenous enzymatic and exogenous non-enzymatic compounds may not be accounted for. In this context, several alternatives have emerged in the last years to measure the oxidative balance of an individual. Yet, there are no unified methodological criteria for the definition of overall antioxidant capacity [61]. A specific oxidative balance index for BC, which accounts for dietary and BC risk factors as well as bioavailability may be useful for further research to clarify the role of antioxidant vitamins and minerals in BC. 

The strengths of this study include its prospective and dynamic design, high overall retention (91%), long follow-up, use of reliable measures to collect dietary habits and other lifestyle information, the ability to adjust for multiple potential confounders, and the complete verification of BC cases by a trained oncologist. However, we acknowledge some limitations of the present study. First, dietary information of vitamins and minerals were assessed with a semi-quantitative FFQ, which may be prone to recall bias; nevertheless, the questionnaire has been repeatedly validated [23,24,25], and we excluded participants with energy intakes outside predefined limits [22]. Second, participants’ diet was evaluated at baseline and may not reflect long-term intake as accurate as repeated measurements of diet during follow-up. Third, we did not take into consideration antioxidant capacity losses caused by cooking methods. Fourth, absence of residual confounding cannot be assumed; however, we adjusted for several potential BC risk factors based on prior literature and previous findings of the SUN cohort. Fifth, the relatively small numbers of BC cases in our cohort may have somewhat limited the statistical power to examine associations. Additionally, multiple testing might explain the presence of significant results in our study, as we examined several compounds; nevertheless, most of the results remained non-significant. Therefore, replication of our findings in larger cohorts should be warranted. Lastly, the participants’ characteristics and homogeneity (Mediterranean middle-age with graduate education) may not represent the general population. In turn, the generalization of our findings should be based on biological mechanisms rather than on statistical representativeness. Moreover, the homogeneity of our cohort increases the reliability of the self-reported data collection and reduces confusion related to education and other socioeconomic factors, increasing the internal validity of these results.

## 5. Conclusions

We found no evidence for a consistent association between intake of vitamins A, C, and E, selenium, and zinc and BC risk either in overall or premenopausal women in this prospective study of the SUN cohort. For postmenopausal women, we observed an inverse association between antioxidant intake and BC risk for vitamin E, but not for the rest of the antioxidants. Our results are in line with most of the findings of cohort studies. Our results, however, should be interpreted with caution, and replication of our findings in larger cohorts should be warranted. Further investigations should consider overall antioxidant capacity concerning BC incidence.

## Figures and Tables

**Table 1:** Baseline characteristics of the female study participants of the Seguimiento Universidad de Navarra (SUN) Project (*n* = 9983).

Characteristics	Total Cohort
N (frequency)	9983
Age, years	35.1 (10.5)
Height, cm	163.6 (6.0)
BMI, kg/m^2^	22.2 (3.0)
Marital status, married, n(%)	4287 (42.9%)
Years of university studies	4.8 (1.3)
Smoking status at baseline, n(%)	
Never smokers	5153 (51.6%)
Current smokers	2304 (23.1%)
Former smokers	2526 (25.3%)
Cumulative smoking habit, packs-years	3.4 (7.0)
Alcohol intake, g/d	4.1 (5.9)
Coffee consumption, >1 cup/d	4601 (46.1%)
Physical activity at baseline, METs-h/week	18.4 (19.1)
TV-watching, h/d	1.6 (1.2)
Oral contraceptive use, n(%)	237 (2.4%)
Multivitamin and/or mineral users at baseline, n(%)	2229 (22.3%)
Self-reported diabetes at baseline, n(%)	102 (1.0%)
*Diet, energy, and nutrients*	
^a^ Adherence to the Mediterranean diet ^a^	3.8 (1.7)
Sugar-sweetened beverage consumption, n(%)	
Never/seldom	3516 (35.2%)
1/month to ≤1 serving/week	4459 (44.7%)
>1 servings/week	2008 (20.1%)
Total energy intake, kcal/d	2279 (571)
Carbohydrate intake, % E	43.2 (7.2)
Protein intake, % E	18.3 (3.2)
Fat intake, % E	37.2 (6.4)
Saturated fatty acids, % E	12.6 (3.1)
Monounsaturated fatty acids, % E	16.1 (3.8)
Polyunsaturated fatty acids, % E	5.1 (1.4)
Family history of breast cancer (first degree), n(%)	1063 (10.6%)
*Reproductive history*	
Obstetric history	
Nulliparous & <25 years	1789 (17.9%)
Nulliparous & ≥25 years	4881 (48.9%)
First pregnancy before 25 years	450 (4.5%)
First pregnancy between 25–29 years	1461 (14.6%)
First pregnancy being 30 years or older	1402 (14.0%)
Lifetime breastfeeding, months	2.3 (4.9)
Age of menarche, n (%)	
≤11 years	2001 (20.0%)
12–13 years	5474 (54.8%)
13–14 years	1703 (17.1%)
≥15 years	805 (8.1%)
Menopausal status at baseline, n (%)	
Premenopausal	9239 (92.5%)
Postmenopausal	744 (7.5%)
^b^ Age at menopause, n (%) ^b^	
Postmenopausal < 50 years	318 (42.7%)
Postmenopausal ≥ 50 years	426 (57.3%)
^b^ Cause of menopause, n (%) ^b^	
Natural	643 (86.4%)
^c^ Induced ^c^	101 (13.6%)
^b^ Hormone replacement therapy, n (%) ^b^	
Ever-use	446 (4.5%)
Duration of use, years	1.4 (2.5)

Note: Mean values and standard deviations, unless otherwise stated. Abbreviations: BMI, body mass index; E, energy; MET, metabolic equivalent. ^a^ Modified Mediterranean adherence score proposed by Trichopoulou et al. [32]. ^b^ Among postmenopausal women. ^c^ Induced menopause included menopause due to surgery/hysterectomy, radiation, medication, or other conditions.

**Table 2:** Dietary vitamin A intake. Hazard Ratio (HR) and 95% Confidence Interval (CI) of breast cancer by tertiles of energy-adjusted dietary vitamin A at baseline among female participants of the SUN cohort.

	Tertiles of Energy-Adjusted of Dietary Vitamin A Intake			
	T1	T2	T3	*P* for Trend
*Overall*				
Intake range, (mcg⁄d)	<1387	1387–2282	>2282	
Median intake, (mcg⁄d)	1033	1747	2984	
No of participants	3328	3328	3327	
Person-years	38,540	37,718	36,740	
Cases	36	31	40	
Incidence rate/10,000 person-years	9.34	821	10.88	
Age-adjusted	1.00 (reference)	0.80 (0.49–1.29)	0.96 (0.61–1.50)	0.998
Model 1	1.00 (reference)	0.77 (0.48–1.26)	0.94 (0.59–1.50)	0.975
Model 2	1.00 (reference)	0.85 (0.51–1.41)	1.07 (0.64–1.77)	0.673
*Premenopausal*				
Intake range, (mcg⁄d)	<1366	1366–2250	>2250	
Median intake, (mcg⁄d)	1017	1719	2944	
No of participants	3077	3077	3076	
Person-years	33,149	32,113	30,580	
Cases	22	20	18	
Incidence rate/10,000 person-years	6.64	6.23	5.89	
Age-adjusted	1.00 (reference)	0.88 (0.48–1.61)	0.78 (0.42–1.45)	0.434
Model 1	1.00 (reference)	0.88 (0.48–1.63)	0.77 (0.40–1.46)	0.423
Model 2	1.00 (reference)	0.95 (0.50–1.80)	0.81 (0.40–1.62)	0.537
*Postmenopausal*				
Intake range, (mcg⁄d)	<1513	1513–2499	>2499	
Median intake, (mcg⁄d)	1153	1924	3249	
No of participants	987	987	987	
Person-years	6307	6860	6844	
Cases	12	10	17	
Incidence rate/10,000 person-years	19.02	14.58	24.84	
Age-adjusted	1.00 (reference)	0.80 (0.35–1.86)	1.35 (0.65–2.84)	0.311
Model 1	1.00 (reference)	0.72 (0.31–1.70)	1.51 (0.70–3.23)	0.178
Model 2	1.00 (reference)	0.78 (0.32–1.90)	1.72 (0.73–4.01)	0.127

Abbreviations: CI, confidence interval; HR, hazard ratio; Ref., reference. -Model 1: crude model additionally adjusted for age of menarche (four categories), age at menopause (three categories), alcohol intake (g/d, continuous), breastfeeding (months, continuous), BMI (kg/m^2^, continuous), height (cm, continuous), hormone replacement therapy (dichotomous) and its duration (years, continuous), obstetric history (five categories), physical activity (metabolic equivalent-h/week, continuous), relatives with history of breast cancer (three categories), smoking habit (package/year, continuous), smoking status (three categories), and years at university (continuous). For premenopausal women, models were not adjusted for age at menopause, hormone replacement therapy, and its duration. For postmenopausal women, models were additionally adjusted for time since recruitment until the beginning of the time at risk (years, continuous). -Model 2: model 1 additionally adjusted for calcium intake (mg/d, continuous), coffee consumption (two categories), fat intake (E%, continuous), Mediterranean diet adherence (points, continuous), sugar-sweetened beverages (three categories), total energy intake (Kcal/d, continuous), TV-watching (hours/d, continuous), and use of supplements (dichotomous).

**Table 3:** Dietary vitamin C intake. Hazard Ratio (HR) and 95% Confidence Interval (CI) of breast cancer by tertiles of energy-adjusted dietary vitamin C at baseline among female participants of the SUN cohort.

	Tertiles of Energy-Adjusted of Dietary Vitamin C Intake			
	T1	T2	T3	*P* for Trend
*Overall*				
Intake range, (mg⁄d)	<219	219–322	>322	
Median intake, (mg⁄d)	168	265	406	
No of participants	3328	3328	3327	
Person-years	38,458	37,754	36,878	
Cases	39	28	40	
Incidence rate/10,000 person-years	10.14	7.41	10.87	
Age-adjusted	1.00 (reference)	0.68 (0.42–1.10)	0.89 (0.57–1.39)	0.756
Model 1	1.00 (reference)	0.70 (0.43–1.15)	0.92 (0.58–1.46)	0.866
Model 2	1.00 (reference)	0.73 (0.43–1.22)	1.00 (0.58–1.71)	0.846
*Premenopausal*				
Intake range, (mg⁄d)	<216	216–317	>317	
Median intake, (mg⁄d)	167	262	401	
No of participants	3077	3077	3076	
Person-years	33,385	31,971	30,485	
Cases	23	18	19	
Incidence rate/10,000 person-years	6.89	5.63	6.23	
Age-adjusted	1.00 (reference)	0.73 (0.44–1.22)	0.82 (0.50–1.33)	0.485
Model 1	1.00 (reference)	0.76 (0.46–1.28)	0.87 (0.53–1.44)	0.652
Model 2	1.00 (reference)	0.76 (0.44–1.31)	0.88 (0.49–1.59)	0.758
*Postmenopausal*				
Intake range, (mg⁄d)	<235	235–348	>348	
Median intake, (mg⁄d)	181	289	432	
No of participants	987	987	987	
Person-years	6016	6914	7081	
Cases	11	12	16	
Incidence rate/10,000 person-years	18.29	17.36	22.60	
Age-adjusted	1.00 (reference)	0.98 (0.43–2.23)	1.28 (0.59–2.77)	0.485
Model 1	1.00 (reference)	1.00 (0.43–2.31)	1.40 (0.63–3.12)	0.368
Model 2	1.00 (reference)	1.13 (0.46–2.74)	1.72 (0.66–4.45)	0.234

Abbreviations: CI, confidence interval; HR, hazard ratio; Ref., reference. -Model 1: crude model additionally adjusted for age of menarche (four categories), age at menopause (three categories), alcohol intake (g/d, continuous), breastfeeding (months, continuous), BMI (kg/m^2^, continuous), height (cm, continuous), hormone replacement therapy (dichotomous) and its duration (years, continuous), obstetric history (five categories), physical activity (metabolic equivalent-h/week, continuous), relatives with history of breast cancer (three categories), smoking habit (package/year, continuous), smoking status (three categories), and years at university (continuous). For premenopausal women, models were not adjusted for age at menopause, hormone replacement therapy and its duration. For menopausal women, models were additionally adjusted for time since recruitment until the beginning of the time at risk (years, continuous). -Model 2: model 1 additionally adjusted for calcium intake (mg/d, continuous), coffee consumption (two categories), fat intake (E%, continuous), Mediterranean diet adherence (points, continuous), sugar-sweetened beverages (three categories), total energy intake (Kcal/d, continuous), TV-watching (hours/d, continuous), and use of supplements (dichotomous).

**Table 4:** Dietary vitamin E intake. Hazard Ratio (HR) and 95% Confidence Interval (CI) of breast cancer by tertiles of energy-adjusted dietary vitamin E at baseline among female participants of the SUN cohort.

	Tertiles of Energy-Adjusted of Dietary Vitamin E Intake			
	T1	T2	T3	*P* for Trend
*Overall*				
Intake range, (mg⁄d)	<5.56	5.56–7.15	>7.15	
Median intake, (mg⁄d)	4.68	6.30	8.87	
No of participants	3328	3328	3327	
Person-years	38,036	37,475	37,4788	
Cases	38	36	33	
Incidence rate/10,000 person-years	9.99	9.61	8.80	
Age-adjusted	1.00 (reference)	0.94 (0.60–1.48)	0.86 (0.54–1.37)	0.525
Model 1	1.00 (reference)	0.94 (0.59–1.48)	0.86 (0.54–1.38)	0.533
Model 2	1.00 (reference)	1.00 (0.62–1.63)	0.92 (0.55–1.54)	0.728
*Premenopausal*				
Intake range, (mg⁄d)	<5.53	5.53–7.13	>7.13	
Median intake, (mg⁄d)	4.66	6.27	8.79	
No of participants	3077	3077	3076	
Person-years	32,260	31,894	31,687	
Cases	17	26	17	
Incidence rate/10,000 person-years	5.27	8.15	5.37	
Age-adjusted	1.00 (reference)	1.00 (0.61–1.64)	0.93 (0.56–1.55)	0.779
Model 1	1.00 (reference)	0.99 (0.60–1.62)	0.95 (0.57–1.58)	0.832
Model 2	1.00 (reference)	1.05 (0.62–1.79)	1.03 (0.59–1.81)	0.924
*Postmenopausal*				
Intake range, (mg⁄d)	<5.63	5.63–7.27	>7.27	
Median intake, (mg⁄d)	4.74	6.41	9.10	
No of participants	987	987	987	
Person-years	6592	6568	6850	
Cases	20	10	9	
Incidence rate/10,000 person-years	30.33	15.22	13.14	
Age-adjusted	1.00 (reference)	0.50 (0.24–1.08)	0.44 (0.20–0.96)	0.042
Model 1	1.00 (reference)	0.47 (0.22–1.01)	0.40 (0.18–0.90)	0.028
Model 2	1.00 (reference)	0.45 (0.20–1.04)	0.35 (0.14–0.86)	0.027

Abbreviations: CI, confidence interval; HR, hazard ratio; Ref., reference. -Model 1: crude model additionally adjusted for age of menarche (four categories), age at menopause (three categories), alcohol intake (g/d, continuous), breastfeeding (months, continuous), BMI (kg/m^2^, continuous), height (cm, continuous), hormone replacement therapy (dichotomous) and its duration (years, continuous), obstetric history (five categories), physical activity (metabolic equivalent-h/week, continuous), relatives with history of breast cancer (three categories), smoking habit (package/year, continuous), smoking status (three categories), and years at university (continuous). For premenopausal women, models were not adjusted for age at menopause, hormone replacement therapy and its duration. For menopausal women, models were additionally adjusted for time since recruitment until the beginning of the time at risk (years, continuous). -Model 2: model 1 additionally adjusted for calcium intake (mg/d, continuous), coffee consumption (two categories), fat intake (E%, continuous), Mediterranean diet adherence (points, continuous), sugar-sweetened beverages (three categories), total energy intake (Kcal/d, continuous), TV-watching (hours/d, continuous), and use of supplements (dichotomous).

**Table 5:** Dietary selenium intake. Hazard Ratio (HR) and 95% Confidence Interval (CI) of breast cancer by tertiles of energy-adjusted dietary selenium at baseline among female participants of the SUN cohort.

	Tertiles of Energy-Adjusted of Dietary Selenium Intake			
	T1	T2	T3	*P* for Trend
*Overall*				
Intake range, (mcg⁄d)	<80.9	80.9–101.1	>101.1	
Median intake, (mcg⁄d)	69.0	90.5	114.5	
No of participants	3328	3328	3327	
Person-years	37,884	37,563	37,553	
Cases	32	26	49	
Incidence rate/10,000 person-years	8.44	6.92	13.05	
Age-adjusted	1.00 (reference)	0.74 (0.44–1.25)	1.26 (0.81–1.97)	0.215
Model 1	1.00 (reference)	0.73 (0.43–1.23)	1.27 (0.81–1.99)	0.209
Model 2	1.00 (reference)	0.76 (0.45–1.29)	1.37 (0.85–2.20)	0.135
*Premenopausal*				
Intake range, (mcg⁄d)	<80.4	80.4–100.3	>100.3	
Median intake, (mcg⁄d)	68.6	90.1	113.8	
No of participants	3077	3077	3076	
Person-years	32,766	31,929	31,147	
Cases	19	17	24	
Incidence rate/10,000 person-years	5.80	5.32	7.71	
Age-adjusted	1.00 (reference)	0.67 (0.38–1.17)	1.18 (0.73–1.90)	0.379
Model 1	1.00 (reference)	0.64 (0.36–1.13)	1.24 (0.77–2.01)	0.268
Model 2	1.00 (reference)	0.65 (0.37–1.16)	1.29 (0.78–2.14)	0.226
*Postmenopausal*				
Intake range, (mcg⁄d)	< 84.5	84.5–105.2	> 105.2	
Median intake, (mcg⁄d)	72.7	93.8	118.2	
No of participants	987	987	987	
Person-years	6360	6663	6988	
Cases	11	8	20	
Incidence rate/10,000 person-years	17.30	12.01	28.62	
Age-adjusted	1.00 (reference)	0.69 (0.28–1.71)	1.68 (0.80–3.51)	0.106
Model 1	1.00 (reference)	0.64 (0.25–1.61)	1.65 (0.78–3.48)	0.115
Model 2	1.00 (reference)	0.68 (0.27–1.75)	1.70 (0.77–3.75)	0.118

Abbreviations: CI, confidence interval; HR, hazard ratio; Ref., reference. -Model 1: crude model additionally adjusted for age of menarche (four categories), age at menopause (three categories), alcohol intake (g/d, continuous), breastfeeding (months, continuous), BMI (kg/m^2^, continuous), height (cm, continuous), hormone replacement therapy (dichotomous) and its duration (years, continuous), obstetric history (five categories), physical activity (metabolic equivalent-h/week, continuous), relatives with history of breast cancer (three categories), smoking habit (package/year, continuous), smoking status (three categories), and years at university (continuous). For premenopausal women, models were not adjusted for age at menopause, hormone replacement therapy and its duration. For menopausal women, models were additionally adjusted for time since recruitment until the beginning of the time at risk (years, continuous). -Model 2: model 1 additionally adjusted for calcium intake (mg/d, continuous), coffee consumption (two categories), fat intake (E%, continuous), Mediterranean diet adherence (points, continuous), sugar-sweetened beverages (three categories), total energy intake (Kcal/d, continuous), TV-watching (hours/d, continuous), and use of supplements (dichotomous).

**Table 6:** Dietary zinc intake. Hazard Ratio (HR) and 95% Confidence Interval (CI) of breast cancer by tertiles of energy-adjusted dietary zinc at baseline among female participants of the SUN cohort.

	Tertiles of Energy-Adjusted of Dietary Zinc Intake			
	T1	T2	T3	*P* for Trend
*Overall*				
Intake range, (mg⁄d)	<12.8	12.8–17.2	>17.2	
Median intake, (mg⁄d)	11.5	14.2	22.9	
No of participants	3328	3328	3327	
Person-years	39,115	37,831	36,053	
Cases	38	35	34	
Incidence rate/10,000 person-years	9.72	9.25	9.43	
Age-adjusted	1.00 (reference)	0.92 (0.58–1.45)	0.92 (0.58–1.46)	0.770
Model 1	1.00 (reference)	0.93 (0.59–1.48)	0.93 (0.58–1.50)	0.815
Model 2	1.00 (reference)	0.98 (0.61–1.58)	1.01 (0.61–1.69)	0.939
*Premenopausal*				
Intake range, (mg⁄d)	<12.8	12.8–17.0	>17.0	
Median intake, (mg⁄d)	11.5	14.1	22.6	
No of participants	3077	3077	3076	
Person-years	33,391	31,851	30,600	
Cases	19	21	20	
Incidence rate/10,000 person-years	5.69	6.59	6.54	
Age-adjusted	1.00 (reference)	0.95 (0.57–1.57)	1.06 (0.65–1.75)	0.729
Model 1	1.00 (reference)	0.99 (0.59–1.65)	1.16 (0.69–1.93)	0.521
Model 2	1.00 (reference)	1.04 (0.62–1.76)	1.26 (0.73–2.19)	0.367
*Postmenopausal*				
Intake range, (mg⁄d)	<12.9	12.9–17.6	>17.6	
Median intake, (mg⁄d)	11.6	14.3	24.7	
No of participants	987	987	987	
Person-years	6406	6618	6987	
Cases	14	12	13	
Incidence rate/10,000 person-years	21.85	18.13	18.61	
Age-adjusted	1.00 (reference)	0.84 (0.39–1.83)	0.85 (0.40–1.82)	0.761
Model 1	1.00 (reference)	0.84 (0.39–1.84)	0.82 (0.37–1.78)	0.680
Model 2	1.00 (reference)	0.89 (0.40–1.99)	0.89 (0.38–2.07)	0.840

Abbreviations: CI, confidence interval; HR, hazard ratio; Ref., reference. -Model 1: crude model additionally adjusted for age of menarche (four categories), age at menopause (three categories), alcohol intake (g/d, continuous), breastfeeding (months, continuous), BMI (kg/m^2^, continuous), height (cm, continuous), hormone replacement therapy (dichotomous) and its duration (years, continuous), obstetric history (five categories), physical activity (metabolic equivalent-h/week, continuous), relatives with history of breast cancer (three categories), smoking habit (package/year, continuous), smoking status (three categories), and years at university (continuous). For premenopausal women, models were not adjusted for age at menopause, hormone replacement therapy and its duration. For menopausal women, models were additionally adjusted for time since recruitment until the beginning of the time at risk (years, continuous). -Model 2: model 1 additionally adjusted for calcium intake (mg/d, continuous), coffee consumption (two categories), fat intake (E%, continuous), Mediterranean diet adherence (points, continuous), sugar-sweetened beverages (three categories), total energy intake (Kcal/d, continuous), TV-watching (hours/d, continuous), and use of supplements (dichotomous).

## Data Availability

The data presented in this study are available on request from the corresponding author.

## Supplementary Materials

The following are available online at https://www.mdpi.com/2076-3921/10/3/340/s1, Table S1: Sources of variability (cumulative R^2^) and main sources (%) of dietary vitamin A, vitamin C, vitamin E, selenium, and zinc intake according to each food included in the FFQ and food groups. Table S2: Sensitivity analyses: luminal breast cancer. Hazard Ratio (HR) and 95% Confidence Interval (CI) of breast cancer by tertiles of energy-adjusted dietary vitamins A, C, and E, selenium, and zinc at baseline among female participants of the SUN cohort (*n* = 9983). Table S3: Sensitivity analyses: excluding participants with a follow-up <2 years. Hazard Ratio (HR) and 95% Confidence Interval (CI) for overall, premenopausal, and postmenopausal breast cancer excluding participants with short period of follow-up (<2 years after entering into the cohort). Table S4: Sensitivity analyses: truncating participants’ follow-up at 10 years. Hazard Ratio (HR) and 95% Confidence Interval (CI) for overall, premenopausal, and postmenopausal breast cancer truncating follow-up after 10 years. Supplemental Table S5: Sensitivity analyses: total (dietary + supplements) intake of vitamins A, C, and E, and zinc. Hazard Ratio (HR) and 95% Confidence Interval (CI) of breast cancer by tertiles of energy-adjusted of total (dietary + supplements) intake of vitamins A, C, and E, and zinc at baseline among female participants of the SUN cohort.
