# Supplementary material for: Dietary Antioxidant Vitamins and Minerals and Breast Cancer Risk: Prospective Results from the SUN Cohort
Source: Antioxidants (Basel). 2021 Feb 24;10(3):340. doi: 10.3390/antiox10030340 (PMC7996327; doi:10.3390/antiox10030340)
Supplement: Supplementary file 1 [file antioxidants-10-00340-s001.pdf]

Supplementary Materials

Dietary Antioxidant Vitamins and Minerals and Breast Cancer Risk: Prospective Results from the SUN Cohort

Cesar I Fernandez-Lazaro, Miguel Ángel Martínez-González, Inmaculada Aguilera-Buenosvinos, Alfredo Gea, Miguel Ruiz-Canela, Andrea Romanos-Nanclares and Estefanía Toledo

Supplemental Table s1: Sources of variability (cumulative R^2) and main sources (%) in dietary vitamin A, vitamin C, vitamin E, selenium, and zinc intake according to each food included in the FFQ and food groups.

Supplemental Table s2: Sensitivity analyses: luminal breast cancer. Hazard Ratio (HR) and 95% Confidence Interval (CI) of luminal breast cancer by tertiles of energy-adjusted dietary vitamins A, C, and E, selenium and zinc at baseline among female participants of the SUN cohort (n= 9,983).

Supplemental Table s3: Sensitivity analyses: excluding participants with a follow-up < 2 years. Hazard Ratio (HR) and 95% Confidence Interval (CI) for overall, premenopausal, and postmenopausal breast cancer excluding participants with short period of follow-up (<2 years after entering into the cohort).

Supplemental Table s4: Sensitivity analyses: truncating participants' follow-up at10 years. Hazard Ratio (HR) and 95% Confidence Interval (CI) for overall, premenopausal, and postmenopausal breast cancer truncating follow-up after 10 years.

Supplemental Table s5: Sensitivity analyses: total (dietary + supplements) intake of vitamins A, C, and E, and zinc. Hazard Ratio (HR) and 95% Confidence Interval (CI) of breast cancer by tertiles of energy-adjusted of total (dietary + supplements) intake of vitamins A, C, and E, and zinc at baseline among female participants of the SUN cohort.

Table s1. Sources of variability (cumulative R^2) and main sources (%) in dietary Vitamin A, vitamin C, vitamin E, selenium, and zinc intake according to each food included in the FFQ and food groups.

Food item / Food group	Cumulative R^2	Percentage (%) of total dietary nutrient intake
Vitamin A		
**Food item**		
Carrots	0.7913	34.70
Chards	0.8909	15.27
Lettuce	0.9441	12.90
Sweet melon	0.9689	4.51
Tomatoes	0.9805	8.09
**Food group**		
Vegetables	0.9587	73.45
Fruits	0.9876	10.49
Dairy products	0.9986	9.18
Fats and oils	0.9995	1.69
Eggs	0.9997	2.16
Vitamin C		
**Food item**		
Peppers	0.3422	18.70
Oranges	0.6468	13.88
Kiwi	0.7654	6.83
Lettuce	0.8094	6.41
Sweet melon	0.8451	3.20
**Food group**		
Vegetables	0.5825	53.20
Fruits	0.9898	36.98
Cereals and legumes	0.9931	1.6
Dairy products	0.9938	2.37
Meat and meat products	0.9940	0.49
Vitamin E		
**Food item**		
Nuts	0.3407	13.20
Sunflower oil	0.6819	7.08
Marzipan	0.7718	4.69
Olive oil	0.8321	13.55
Peppers	0.8624	5.53
**Food group**		
Fats and oils	0.4284	28.04
Nuts	0.7871	14.08
Pastries	0.9066	10.41
Vegetables	0.9432	9.48
Cereals and legumes	0.9549	6.94
Selenium		
**Food item**		
Eggs	0.2808	26.36

White bread	0.4958	14.84
White fish	0.6812	11.06
Whole bread	0.8151	4.91
Fatty fish	0.9178	10.88
**Food group**		
Cereals and legumes	0.4041	21.49
Fish and seafood	0.7089	26.45
Eggs	0.9405	26.36
Meat and meat products	0.9701	10.47
Dairy products	0.9916	8.03
Zinc		
**Food item**		
Low-fat yogurt	0.7680	17.48
Beef	0.8062	8.61
Cream cheese	0.8340	4.59
White bread	0.8559	6.86
Whole bread	0.8813	2.79
**Food group**	0.8939	2.53
Dairy products	0.8194	30.96
Meat and meat products	0.8993	26.02
Cereals and legumes	0.9581	15.86
Vegetables	0.9800	9.90
Fruits	0.9869	4.73

Table s2. Sensitivity analyses: luminal breast cancer. Hazard Ratio (HR) and 95% Confidence Interval (CI) of luminal breast cancer by tertiles of energy-adjusted dietary vitamins A, C, and E, selenium and zinc at baseline among female participants of the SUN cohort (n= 9,983).

Variables	Tertiles of energy-adjusted of dietary intake			*P* for trend
	T1	T2	T3	
Vitamin A				
Intake range, (mcg⁄d)	< 1,387	1,387-2,282	>2,282	
Mean intake, (mcg⁄d)	1,033	1,747	2,984	
No of participants	3,328	3,328	3,327	
Person-years	38,540	37,718	36,740	
Cases	16	16	25	
Incidence rate/10,000 person-years	4,15	4,24	6,80	
Age-adjusted	1.00 (reference)	0.93 (0.46-1.86)	1.35 (0.72-2.54)	0.264
Model 1	1.00 (reference)	0.90 (0.45-1.82)	1.38 (0.73-2.64)	0.229
Model 2	1.00 (reference)	0.93 (0.45-1.91)	1.43 (0.71-2.89)	0.229
Vitamin C				
Intake range, (mg⁄d)	< 219	219-322	>322	
Mean intake, (mg⁄d)	168	265	406	
No of participants	3,328	3,328	3,327	
Person-years	38,458	37,754	36,787	
Cases	21	14	22	
Incidence rate/10,000 person-years	5.46	3.71	5.98	
Age-adjusted	1.00 (reference)	0.63 (0.32-1.24)	0.92 (0.50-1.68)	0.922
Model 1, (mg⁄d)	1.00 (reference)	0.65 (0.33-1.29)	0.98 (0.53-1.83)	0.910
Model 2, (mg⁄d)	1.00 (reference)	0.59 (0.29-1.21)	0.88 (0.42-1.83)	0.897
Vitamin E				
Intake range, (mg⁄d)	< 5.56	5.56-7.15	>7.15	
Mean intake, (mg⁄d)	4.68	6.30	8.87	
No of participants	3,328	3,328	3,327	
Person-years	38,036	37,475	37,488	
Cases	21	18	18	
Incidence rate/10,000 person-years	5.52	4.80	4.80	
Age-adjusted	1.00 (reference)	0.85 (0.46-1.60)	0.85 (0.45-1.60)	0.644
Model 1	1.00 (reference)	0.88 (0.47-1.65)	0.89 (0.47-1.67)	0.730
Model 2	1.00 (reference)	0.93 (0.47-1.85)	1.03 (0.51-2.09)	0.895
Selenium				
Intake range, (mcg⁄d)	< 80.9	80.9-101.1	>101.1	
Mean intake, (mcg⁄d)	69.0	90.5	114.5	
No of participants	3,328	3,328	3,327	
Person-years	3.96	3.99	7.19	
Cases	15	15	27	
Incidence rate/10,000 person-years	3.96	3.99	7.19	
Age-adjusted	1.00 (reference)	0.92 (0.45-1.87)	1.49 (0.79-2.80)	0.169
Model 1	1.00 (reference)	0.90 (0.44-1.86)	1.52 (0.80-2.88)	0.153
Model 2	1.00 (reference)	0.89 (0.43-1.85)	1.58 (0.81-3.11)	0.136

Zinc				
Intake range, (mg⁄d)	< 12.8	12.8-17.2	>17.2	
Mean intake, (mg⁄d)	11.5	14.2	22.9	
No of participants	3,328	3,328	3,327	
Person-years	39,115	37,831	36,053	
Cases	19	16	22	
Incidence rate/10,000 person-years	4.86	4.22	6.10	
Age-adjusted	1.00 (reference)	0.84 (0.43-1.63)	1.19 (0.64-2.21)	0.413
Model 1	1.00 (reference)	0.85 (0.43-1.66)	1.27 (0.68-2.40)	0.311
Model 2	1.00 (reference)	0.81 (0.41-1.62)	1.16 (0.58-2.30)	0.465

Abbreviations: CI, confidence interval; HR, hazard ratio; Ref., reference.
- Model 1: crude model additionally adjusted for age of menarche (four categories), age at menopause (three categories), alcohol intake (g/d, continuous), breast-feeding (months, continuous), BMI (kg/m^2, continuous), height (cm, continuous), hormone replacement therapy (dichotomous), obstetric history (five categories), physical activity (metabolic equivalent-h/week, continuous), relatives with history of breast cancer (three categories), smoking habit (package/year, continuous), smoking status (three categories), and years at university (continuous).
- Model 2: model 1 additionally adjusted for calcium intake (mg/d, continuous), coffee consumption (two categories), fat intake (E%, continuous), Mediterranean diet adherence (points, continuous), sugar-sweetened beverages (three categories), total energy intake (Kcal/d, continuous), TV-watching (hours/d, continuous), and use of supplements (dichotomous).

Variable	*Overall*				*Premenopausal*				*Postmenopausal*			
	**Tertiles of energy-adjusted of dietary intakes**			*P* for trend	**Tertiles of energy-adjusted of dietary intakes**			*P* for trend	**Tertiles of energy-adjusted of dietary intakes**			*P* for trend
	**T1**	**T2**	**T3**		**T1**	**T2**	**T3**		**T1**	**T2**	**T3**	
Vitamin A (mcg/d)												
No of participants	3,304	3,303	3,303		3,057	3,057	3,057		983	982	982	
Person-years	31,897	31,055	30,113		33,095	32,013	30,544		6,292	6,854	6,821	
Cases	34	29	37		21	18	16		11	10	17	
Incidence rate/10,000 person-years	10.66	9.34	12.29		6.35	5.62	5.24		17.48	14.59	24.92	
Adjusted Model	1.00 (reference)	0.86 (0.51-1.44)	1.07 (0.63-1.80)	0.695	1.00 (reference)	0.90 (0.46-1.76)	0.77 (0.37-1.60)	0.484	1.00 (reference)	0.85 (0.34-2.11)	1.95 (0.82-4.65)	0.075
Vitamin C (mg/d)												
No of participants	3,304	3,303	3,303		3,057	3,057	3,057		983	982	982	
Person-years	31,824	31,088	30,154		33,305	31,905	30,443		5,995	6,918	7,054	
Cases	38	27	35		21	18	16		11	11	16	
Incidence rate/ 10,000 person-years	11.94	8.69	11.61		6.31	5.64	5.26		18.35	15.90	22.68	
Adjusted Model	1.00 (reference)	0.71 (0.42-1.20)	0.90 (0.51-1.57)	0.825	1.00 (reference)	0.80 (0.46-1.39)	0.82 (0.44-1.52)	0.571	1.00 (reference)	1.07 (0.43-2.65)	1.81 (0.70-4.72)	0.184
Vitamin E (mg/d)												
No of participants	3,304	3,303	3,303		3,057	3,057	3,057		983	982	982	
Person-years	31,346	30,849	30,870		32,170	31,869	31,614		6,575	6,553	6,838	
Cases	36	34	30		15	25	15		20	9	9	
Incidence rate/10,000 person-years	11.48	11.02	9.72		4.66	7.84	4.74		30.42	13.73	13.16	

Adjusted Model	1.00 (reference)	1.01 (0.61-1.67)	0.91 (0.53-1.56)	0.711	1.00 (reference)	1.11 (0.64-1.93)	1.04 (0.58-1.87)	0.938	1.00 (reference)	**0.39 (0.17-0.93)**	**0.33 (0.13-0.82)**	**0.023**
Selenium (mcg⁄d)												
No of participants	3,304	3,303	3,303		3,057	3,057	3,057		983	982	982	
Person-years	31,262	30,852	30,950		32,701	31,796	31,156		6,349	6,658	6,960	
Cases	29	25	46		16	17	22		10	8	20	
Incidence rate/10,000 person-years	9.28	8.10	14.86		4.89	5.35	7.06		15.75	12.01	28.73	
Adjusted Model	1.00 (reference)	0.80 (0.46-1.38)	1.46 (0.89-2.41)	0.090	1.00 (reference)	0.74 (0.41-1.33)	1.37 (0.80-2.33)	0.178	1.00 (reference)	0.72 (0.27-1.88)	1.89 (0.84-4.25)	0.071
Zinc (mg⁄d)												
No of participants	3,304	3,303	3,303		3,057	3,057	3,057		983	982	982	
Person-years	32,470	31,218	29,377		33,314	31,848	30,491		6,403	6,598	6,966	
Cases	35	32	33		18	18	19		13	12	13	
Incidence rate/10,000 person-years	10.78	10.25	11.23		5.40	5.65	6.23		20.3	18.2	18.7	
Adjusted Model	1.00 (reference)	0.96 (0.58-1.59)	1.07 (0.63-1.81)	0.731	1.00 (reference)	0.99 (0.57-1.72)	1.32 (0.75-2.32)	0.266	1.00 (reference)	0.96 (0.42-2.18)	0.98 (0.41-2.31)	0.979

Abbreviations: CI, confidence interval; HR, hazard ratio; Ref., reference.

- Adjusted model: crude model additionally adjusted for age of menarche (four categories), age at menopause (three categories), alcohol intake (g/d, continuous), BMI (kg/m^2, continuous), calcium intake (continuous), coffee consumption (two categories), fat intake (E%, continuous), height (cm, continuous), hormone replacement therapy (dichotomous) and its duration (continuous), Mediterranean diet adherence (continuous), months of breast-feeding (continuous), obstetric history (five categories), physical activity (metabolic equivalent-h/week, continuous), relatives with history of breast cancer (three categories), smoking habit (continuous), smoking status (three categories), sugar-sweetened beverages (three categories), total energy intake (Kcal/d, continuous), TV-watching (continuous), use of supplements (dichotomous), and years at university (continuous). For premenopausal women, models were not adjusted for age at menopause, hormone replacement therapy and its duration. For menopausal women, models were additionally adjusted for time since recruitment until the beginning of the time at risk (years, continuous).

Variable	*Overall*				*Premenopausal*				*Postmenopausal*			
	**Tertiles of energy-adjusted of dietary intakes**			*P* for trend	**Tertiles of energy-adjusted of dietary intakes**			*P* for trend	**Tertiles of energy-adjusted of dietary intakes**			*P* for trend
	**T1**	**T2**	**T3**		**T1**	**T2**	**T3**		**T1**	**T2**	**T3**	
Vitamin A (mcg⁄d)												
No of participants	3,328	3,328	3,327		3,077	3,077	3,076		723	723	723	
Person-years	28,950	28,788	28,458		25,746	25,384	24,677		3,912	4,187	4,124	
Cases	23	26	30		14	18	15		8	8	12	
Incidence rate/10,000 person-years	7.94	9.03	10.54		5.44	7.09	6.08		20.45	19.11	29.10	
Adjusted Model	1.00 (reference)	1.10 (0.61-1.99)	1.20 (0.65-2.20)	0.570	1.00 (reference)	1.42 (0.68-2.97)	1.08 (0.48-2.44)	0.989	1.00 (reference)	0.92 (0.32-2.59)	1.72 (0.62-4.80)	0.230
Vitamin C (mg⁄d)												
No of participants	3,328	3,328	3,327		3,077	3,077	3,076		723	723	723	
Person-years	28,923	28,768	58,505		25,895	25,349	24,563		3,739	4,141	4,343	
Cases	23	20	36		15	13	19		6	12	10	
Incidence rate/ 10,000 person-years	7.95	6.95	12.63		5.79	5.13	7.73		16.05	28.98	23.03	
Adjusted Model	1.00 (reference)	0.73 (0.43-1.22)	1.00 (0.58-1.71)	0.846	1.00 (reference)	0.76 (0.44-1.31)	0.88 (0.49-1.59)	0.758	1.00 (reference)	1.74 (0.69-4.39)	2.04 (0.74-5.64)	0.190
Vitamin E (mg⁄d)												
No of participants	3,328	3,328	3,327		3,077	3,077	3,076		723	723	723	
Person-years	28,903	28,567	28,365		25,529	25,257	25,021		3,983	4,029	4,211	
Cases	31	24	24		16	17	14		15	6	7	
Incidence rate/10,000 person-years	10.73	8.37	8.38		6.27	6.73	5.60		37.66	14.89	16.62	

Adjusted Model	1.00 (reference)	1.00 (0.62-1.63)	0.92 (0.55-1.54)	0.728	1.00 (reference)	1.05 (0.62-1.79)	1.03 (0.59-1.81)	0.924	1.00 (reference)	0.47 (0.10-1.07)	**0.42 (0.18-0.98)**	0.080
Selenium (mcg⁄d)												
No of participants	3,328	3,328	3,327		3,077	3,077	3,076		723	723	723	
Person-years	28,755	28,653	28,787		25,588	25,257	24,962		3,971	4,014	4,237	
Cases	24	21	34		15	14	18		8	7	13	
Incidence rate/10,000 person-years	8.35	7.33	11.81		5.86	5.54	7.21		20.14	17.43	30.68	
Adjusted Model	1.00 (reference)	0.76 (0.45-1.29)	1.37 (0.85-2.20)	0.135	1.00 (reference)	0.65 (0.37-1.16)	1.29 (0.78-2.14)	0.226	1.00 (reference)	0.65 (0.25-1.67)	1.40 (0.62-3.18)	0.323
Zinc (mg⁄d)												
No of participants	3,328	3,328	3,327		3,077	3,077	3,076		723	723	723	
Person-years	29,172	28,708	28,316		25,867	25,124	24,816		3,943	3,966	4,313	
Cases	31	22	26		15	14	18		12	7	9	
Incidence rate/10,000 person-years	10.63	7.66	9.18		5.80	5.57	7.25		30.43	17.65	20.87	
Adjusted Model	1.00 (reference)	0.98 (0.61-1.58)	1.01 (0.61-1.69)	0.939	1.00 (reference)	1.04 (0.62-1.76)	1.26 (0.73-2.19)	0.367	1.00 (reference)	0.79 (0.33-1.87)	0.94 (0.39-2.23)	0.962

Abbreviations: CI, confidence interval; HR, hazard ratio; Ref., reference.

- Adjusted model: crude model additionally adjusted for age of menarche (four categories), age at menopause (three categories), alcohol intake (g/d, continuous), BMI (kg/m^2, continuous), calcium intake (continuous), coffee consumption (two categories), fat intake (E%, continuous), height (cm, continuous), hormone replacement therapy (dichotomous) and its duration (continuous), Mediterranean diet adherence (continuous), months of breast-feeding (continuous), obstetric history (five categories), physical activity (metabolic equivalent-h/week, continuous), relatives with history of breast cancer (three categories), smoking habit (continuous), smoking status (three categories), sugar-sweetened beverages (three categories), total energy intake (Kcal/d, continuous), TV-watching (continuous), use of supplements (dichotomous), and years at university (continuous). For premenopausal women, models were not adjusted for age at menopause, hormone replacement therapy and its duration. For menopausal women, models were additionally adjusted for time since recruitment until the beginning of the time at risk (years, continuous).

Table s5. Sensitivity analyses: total (dietary + supplements) intake of vitamins A, C, and E, and zinc. Hazard Ratio (HR) and 95% Confidence Interval (CI) of breast cancer by tertiles of energy-adjusted of total (dietary + supplements) intake of vitamins A, C, and E, and zinc at baseline among female participants of the SUN cohort.

Variables	Tertiles of energy-adjusted of total (dietary + supplements) intake			P for trend
	T1	T2	T3	
Vitamin A				
Overall				
Adjusted Model	1.00 (reference)	0.83 (0.51-1.38)	1.05 (0.63-1.74)	0.715
Premenopausal				
Adjusted Model	1.00 (reference)	1.09 (0.58-2.05)	0.85 (0.41-1.73)	0.580
Postmenopausal				
Adjusted Model	1.00 (reference)	0.57 (0.23-1.44)	1.69 (0.74-3.85)	0.096
Vitamin C				
Overall				
Adjusted Model	1.00 (reference)	0.82 (0.50-1.36)	0.93 (0.54-1.61)	0.877
Premenopausal				
Adjusted Model	1.00 (reference)	0.71 (0.41-1.23)	0.83 (0.46-1.49)	0.621
Postmenopausal				
Adjusted Model	1.00 (reference)	1.40 (0.57-3.41)	2.00 (0.76-5.29)	0.157
Vitamin E				
Overall				
Adjusted Model	1.00 (reference)	0.84 (0.52-1.38)	0.83 (0.50-1.39)	0.533
Premenopausal				
Adjusted Model	1.00 (reference)	0.99 (0.58-1.70)	0.98 (0.56-1.71)	0.947
Postmenopausal				
Adjusted Model	1.00 (reference)	0.50 (0.22-1.15)	0.45 (0.19-1.08)	0.092
Zinc				
Overall				
Adjusted Model	1.00 (reference)	1.07 (0.66-1.73)	1.09 (0.65-1.83)	0.770
Premenopausal				
Adjusted Model	1.00 (reference)	1.18 (0.70-2.00)	1.33 (0.76-2.33)	0.349
Postmenopausal				
Adjusted Model	1.00 (reference)	0.81 (0.36-1.85)	0.95 (0.41-2.17)	0.959

Abbreviations: CI, confidence interval; HR, hazard ratio; Ref., reference.

- Adjusted model: crude model additionally adjusted for age of menarche (four categories), age at menopause (three categories), alcohol intake (g/d, continuous), BMI (kg/m^2, continuous), calcium intake (continuous), coffee consumption (two categories), fat intake (E%, continuous), height (cm, continuous), hormone replacement therapy (dichotomous) and its duration (continuous), Mediterranean diet adherence (continuous), months of breast-feeding (continuous), obstetric history (five categories), physical activity (metabolic equivalent-h/week, continuous), relatives with history of breast cancer (three categories), smoking habit (continuous), smoking status (three categories), sugar-sweetened beverages (three categories), total energy intake (Kcal/d, continuous), TV-watching (continuous), and years at university (continuous). For premenopausal women, models were not adjusted for age at menopause, hormone replacement therapy and its duration. For postmenopausal women, models were additionally adjusted for time since recruitment until the beginning of the time at risk (years, continuous).
